# Synaptic branch stability is mediated by non-enzymatic functions of MEC-17/αTAT1 and ATAT-2

**DOI:** 10.1038/s41598-022-18333-2

**Published:** 2022-08-17

**Authors:** Jean-Sébastien Teoh, Amruta Vasudevan, Wenyue Wang, Samiksha Dhananjay, Gursimran Chandhok, Roger Pocock, Sandhya P. Koushika, Brent Neumann

**Affiliations:** 1 Neuroscience Program, Department of Anatomy and Developmental Biology, Monash Biomedicine Discovery Institute, Monash University, Melbourne, VIC 3800 Australia; 2 Department of Biological Sciences, Tata Institute of Fundamental Research, Mumbai, India; 3 Development and Stem Cells Program, Department of Anatomy and Developmental Biology, Monash Biomedicine Discovery Institute, Monash University, Melbourne, VIC 3800 Australia

**Keywords:** Development of the nervous system, Genetics of the nervous system

## Abstract

Microtubules are fundamental elements of neuronal structure and function. They are dynamic structures formed from protofilament chains of α- and β-tubulin heterodimers. Acetylation of the lysine 40 (K40) residue of α-tubulin protects microtubules from mechanical stresses by imparting structural elasticity. The enzyme responsible for this acetylation event is MEC-17/αTAT1. Despite its functional importance, however, the consequences of altered MEC-17/αTAT1 levels on neuronal structure and function are incompletely defined. Here we demonstrate that overexpression or loss of MEC-17, or of its functional paralogue ATAT-2, causes a delay in synaptic branch extension, and defective synaptogenesis in the mechanosensory neurons of *Caenorhabditis elegans*. Strikingly, by adulthood, the synaptic branches in these animals are lost, while the main axon shaft remains mostly intact. We show that MEC-17 and ATAT-2 regulate the stability of the synaptic branches largely independently from their acetyltransferase domains. Genetic analyses reveals novel interactions between both *mec-17* and *atat-2* with the focal adhesion gene *zyx-1/Zyxin*, which has previously been implicated in actin remodelling. Together, our results reveal new, acetylation-independent roles for MEC-17 and ATAT-2 in the development and maintenance of neuronal architecture.

## Introduction

Maintenance of neuronal structure over the lifetime of an animal is essential for a fully functional and adaptable nervous system. The highly polarized nature of neurons, with axons typically extending magnitudes larger than the size of the cell body, makes them particularly susceptible to the mechanical stress associated with normal animal movement. The ability of neurons to resist these stressors is mediated by the cytoskeleton^1^. Major structural and functional components of the cytoskeleton are the microtubules, which are highly conserved cylindrical structures assembled from chains of α- and β-tubulin heterodimers termed protofilaments^2^. Microtubules are dynamic structures, cycling through phases of polymerization and depolymerization^3,4^. They are essential for neuronal development, structure, and function, providing platforms for intracellular transport and organelle positioning, as well as the scaffolds for signaling molecules^3^.

Previous research has provided strong links between microtubule dynamics and maintenance of neuronal structure^5^. In particular, the *C. elegans* posterior lateral microtubule (PLM) neurons have provided a productive model for defining the cellular mechanisms required for maintaining neuronal branches and synapses. These neurons extend a long axon anteriorly to the mid-body region from which a single branch elongates ventrally to synapse with other neurons in the ventral nerve cord^6^. Chen et al*.* demonstrated the importance of MEC-12/α-tubulin and MEC-7/β-tubulin for the maintenance of the synaptic branches in these neurons, with loss-of-function alleles inducing post-developmental branch loss^7^. These defects were dependent on the microtubule-associated RhoGEF, RHGF-1, activating the dual leucine-zipper kinase DLK-1 (a mitogen-activated protein kinase (MAP) triple kinase activated by microtubule disruptions)^7,8^. DLK-1 levels are controlled by the E3 ubiquitin ligase RPM-1/Phr/Pam/Highwire^9^ during synaptogenesis in the PLM neurons^10,11^. Moreover, DLK-1 also modulates the function of microtubule minus-end binding and stabilizing protein PTRN-1/Patronin/CAMSAP3^12^ in PLM synaptogenesis^13^. Overall, these studies highlight the importance of intact and stable microtubules for both synaptogenesis and the maintenance of synaptic branches. However, the role of microtubule post-translational modifications in these processes remains unclear.

Microtubules are subjected to a wide-range of post-translational modifications that affect their organization, dynamics, and cellular interactions^14,15^. The acetylation of α-tubulin lysine-40 (K40) is unique amongst these modifications because it occurs inside the microtubule lumen^16,17^. K40 acetylation was long considered a passive marker of stable microtubules. However, more precise functional insights into this modification were revealed in 2017: K40 acetylation weakens the interactions between protofilaments, increasing microtubule elasticity and providing resistance against mechanical stress^18,19^. Thus, K40 acetylation imparts an important protective role against the constant mechanical forces applied to cells during normal animal behaviour in order to maintain microtubule (and neuronal) integrity. This protective role is particularly important for highly polarized cells like neurons, which frequently extend neurites for magnitudes larger than the size of the soma.

The enzyme responsible for K40 acetylation is α-tubulin acetyltransferase 1 (αTAT1)^20,21^. Loss of the αTAT1 protein in *C. elegans* (termed MEC-17) and its proposed functional paralogue ATAT-2, disorganizes the microtubule network, reducing protofilament numbers and overall microtubule numbers^22,23^. However, recent studies have revealed differences in the functions of MEC-17 and ATAT-2, with ATAT-2 being the predominant acetylator in *C. elegans* neurons^21,23,24^. K40 acetylation is associated with numerous cellular functions, including cell migration and adhesion^25–27^, microtubule-based trafficking^28–30^, autophagy^31^, modulation of kinase signaling^32^, as well as neuronal development and function^21,23,33–36^. Intriguingly, MEC-17/αTAT1 also has enzyme-independent functions, which include its interaction with microtubules, and in the elongation and maintenance of neuronal structure^23,24,37^.

Our previous research demonstrated that MEC-17 functions independently from α-tubulin acetylation as a protective factor in the *C. elegans* mechanosensory neurons^24^. The absence of MEC-17 caused spontaneous, adult-onset axonal degeneration due to severe disruptions in neuronal microtubule structure^22–24^. To determine if the overexpression of MEC-17 could be used to protect against other genetic or environmental neurodegenerative insults, we generated *C. elegans* strains that overexpress MEC-17 specifically in the mechanosensory neurons. Surprisingly, far from being a protective factor, we show that overexpression of MEC-17 induces specific loss of the synaptic branches of the PLM neurons. Our data indicate that this phenotype is independent from MEC-17 acetyltransferase activity. Moreover, we find that loss of MEC-17 or disruption of ATAT-2 also results in the loss of PLM synaptic branches. Finally, we demonstrate that MEC-17 and ATAT-2 function together with the focal adhesion protein ZYX-1/Zyxin to preserve these synapses. Overall, our data establish novel roles for MEC-17/ATAT1 and ATAT-2 in synaptic development and synaptic branch stability.

## Results

### Correct levels of MEC-17 and ATAT-2 are required to maintain synaptic branches

**Figure 1:**
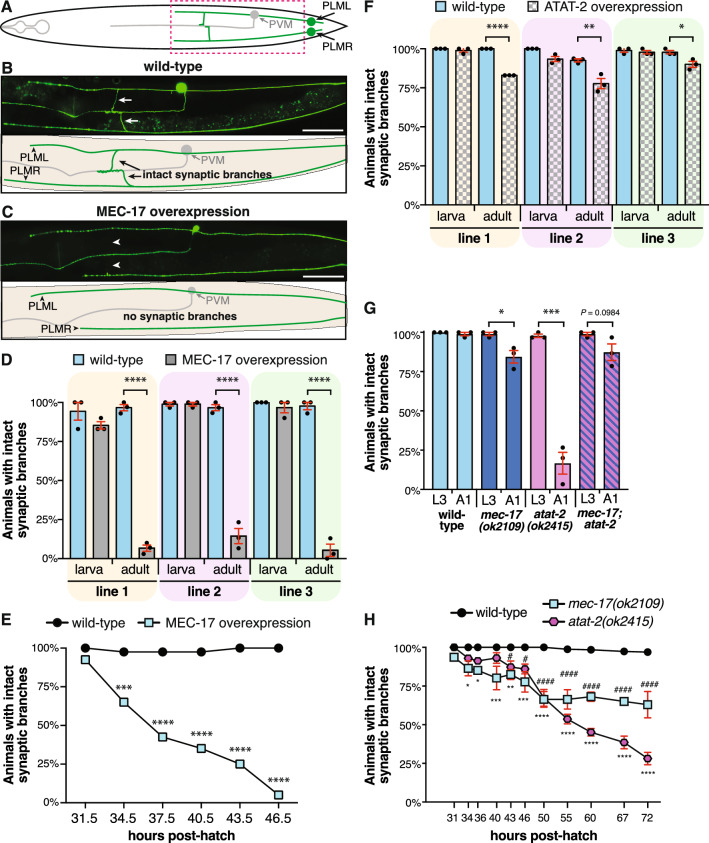
Overexpression and loss of MEC-17 and ATAT-2 disrupts the PLM synaptic branches. (**a**) Schematic representation of a ventral view of the PLM (green) and PVM neurons (gray). Dashed box highlights the approximate location of the regions imaged in (**b,c**). (**b**) Image and schematic showing the intact PLM synaptic branches of a wild-type animal carrying the *zdIs5(Pmec-4::GFP)* transgene. Arrows point to the synaptic branches of PLM left (PLML) and PLM right (PLMR). (**c**) Image and schematic of an animal with overexpression of MEC-17 in which the synaptic branches of both PLML and PLMR are disrupted. Arrowheads point the expected position of the lost branches. Scale bars represent 50 µm. (**d**) Quantification of the number of animals with intact synaptic branches at L3 (larva) or A1 (adult) stages. Three independent transgenic strains are shown (gray bars) compared to their non-transgenic siblings (wild-type, blue bars). Bars show mean ± SE; symbols show the mean of three-independent experiments, each with n ≥ 30 (total n ≥ 90). (**e**) Quantification of the number of animals with an intact synaptic branch across the L4 stage of development in animals carrying the same transgene as line 3 in (**d**) (*cjnEx036(Pmec-4::mec-17)*; blue squares) compared to their non-transgenic siblings (wild-type, black circles). Different groups of animals were analyzed for each time-point; n ≥ 36 for each time-point. (**f**) Quantification of the number of animals with an intact synaptic branch at L3 (larva) or A1 (adult) stages. Three independent transgenic strains with overexpression of ATAT-2 (gray bars) or their non-transgenic siblings (wild-type, blue bars) are shown. Bars show mean ± SE; symbols show the mean of three-independent experiments, each with n ≥ 28 (total n ≥ 89). (**g**) Quantification of the number of animals with intact synaptic branches in *mec-17(ok2109)* and *atat-2(ok2415)* single and double mutant backgrounds at both L3 and A1 stages. Bars show mean ± SE; symbols show the mean of three-independent experiments, each with n ≥ 29 (total n ≥ 90). (**h**) Quantification of the number of animals with intact synaptic branches across different time-points of development in wild-type animals compared to those lacking *mec-17* or *atat-2*. Different groups of animals were analyzed for each time-point; n ≥ 20 for each time-point (total n ≥ 72). *P* values * < 0.05, ** < 0.01, *** < 0.001, **** < 0.0001 from one-way ANOVA with Tukey’s post-hoc tests (**d,f**), Fisher’s exact test (**e**) or unpaired *t*-tests (**g**).

Our previous study revealed that MEC-17 protects the six mechanosensory neurons of *C. elegans* from spontaneous axonal degeneration^24^. As such, we hypothesized that its overexpression might be beneficial in preventing axonal degeneration induced by other genetic or environmental insults. In order to test this, we generated transgenic strains with MEC-17 explicitly overexpressed in the mechanosensory neurons (*Pmec-4::mec-17*), the only cells in which it is normally expressed. To our surprise and in opposition to our hypothesis, overexpression of MEC-17 caused the specific loss of the PLM synaptic branches while leaving the main axon shafts intact (Fig. 1a–d and S1a–d). During normal *C. elegans* development, the PLM synaptic branches form during the first larval stage (L1) and are preserved throughout life^11^. Analysis of larval stage 3 (L3) animals overexpressing MEC-17 revealed that the synaptic branches were present during development. However, by early adulthood (1-day-old adults, A1) the vast majority of synaptic branches in these animals were lost (Fig. 1d). We consistently observed thinning of the PLM branches in animals overexpressing MEC-17, as well as remnants of the branch emanating from the main axon shaft, the pre-synaptic side or both (Fig. S1a–d). These observations suggest that the branch may physically break in these animals rather than undergoing a retraction process. Despite repeated attempts to capture a branch loss event with time-lapse microscopy, we were unable to do so. As imaging requires the animals to be immobilized, it is possible that this inhibited the phenotype by suppressing the mechanical strain associated with normal movement (see below and Fig. S2a,b).

To define when the branch loss occurs, we analyzed animals at three-hour time intervals across the final larval stage (L4) of development, which extends from 31.5–33.5 to 44–46 h post-hatching^38^. At the earliest time point (31.5 h), MEC-17 overexpression caused no detectable defects (Fig. 1e). At each subsequent time point, however, we observed a progressive loss of the synaptic branches, with less than 50% of animals having intact synaptic branches at 37.5 h and only 5% at the final 46.5 h time point. This final level of defect is consistent with that observed in adults (5%, Fig. 1d, line 3), demonstrating that the overexpression of MEC-17 leads to loss of the PLM synaptic branches during the L4 stage of development. As *mec-17* is strongly expressed in the PLM neurons from late embryonic stages through to adulthood^39^, it is unlikely that any temporal changes in expression account for the specific loss of branches at the L4 stage.

MEC-17 has been reported to function redundantly with the ATAT-2 protein in the acetylation of α-tubulin^20,21^. To determine if correct levels of ATAT-2 are also necessary for the maintenance of the PLM synaptic branches, we generated strains overexpressing ATAT-2 specifically in these neurons (*Pmec-4::atat-2*). The relative increases in *atat-2* transcript levels in these strains were comparable with the increases in *mec-17* levels observed in the *mec-17* overexpression strains (Fig. S1e,f). Similar to the phenotypes observed when MEC-17 was overexpressed, we observed no defect at the L3 stage, but a significant proportion of animals overexpressing ATAT-2 displayed disrupted synaptic branches in adulthood (Fig. 1f). However, whereas MEC-17 overexpression led to synaptic defects in more than 85% of animals (Fig. 1d), ATAT-2 overexpression had a more modest effect, with a 10–22% decrease observed across three independent transgenic lines (Fig. 1f).

Although the overexpression of α-tubulin acetyltransferases did not present a viable strategy for preventing axon degeneration, our data provide further support for the role for these enzymes in the maintenance of the PLM synaptic branch^40^. To further confirm this function, we studied null alleles of *mec-17* and *atat-2*^41^. Interestingly, this phenocopied the overexpression studies, with the PLM synaptic branch lost in a significant proportion of adult animals (Fig. 1g). Loss of *atat-2* caused the stronger phenotype, with only 17% of adults possessing intact synaptic branches, compared to 84% of adults in the *mec-17* loss-of-function strain. These defects were partially rescued by the introduction of wild-type copies of MEC-17 (35.78% increase) or ATAT-2 (31% increase) into the PLM neurons of the respective mutant backgrounds (Fig. S1g), indicating predominant cell-autonomous functions for these molecules.

Analysis of double mutant animals revealed that the loss of *mec-17* completely suppressed the stronger *atat-2* mutant phenotype, with 87% of adult animals possessing intact synaptic branches (Fig. 1g). These findings may be driven by the stronger mechanosensation defect in *mec-17* animals^21,23^, causing reduced locomotion and as a result, lessening the movement-induced mechanical stress encountered by their neurons. To test this hypothesis, we treated animals with RNAi-mediated knockdown of *unc-54*, which encodes a myosin heavy chain required for locomotion^42,43^. As shown in Fig. S2a, knockdown of *unc-54* strongly suppressed the synaptic branch loss phenotype observed in animals with overexpression of *mec-17* or carrying the *atat-2* mutation. We also found that paralyzing *atat-2* mutant animals with an anesthetic had a similar effect (Fig. S2b). These data mirror our previous study that found the axonal degeneration associated with loss of *mec-17* can be suppressed by paralysis^24^, and indicate that synaptic branch stability can be modulated by movement.

We next temporally compared the branch loss in *mec-17* and *atat-2* mutants. As shown in Fig. 1h, *mec-17* mutants first began losing synaptic branches 34 h after hatching. By 50 h post-hatching, the highest level of branch loss was seen, with levels remaining relatively constant across the remainder of the 72 h period of analysis. The absence of *atat-2* induced a low level of branch loss from the 43 h time point before a sharp increase was observed between the 50 and 72 h time points. Additionally, we questioned if the loss of the synaptic branch was specific to the L4 stage. As movement appeared to induce the synaptic branch loss, we decided to treat the animals with anesthetic and immobilise them prior to and during branch loss. Anesthetising the animals at specific time-points during the L4 stage (39–45 h or 48–54 h post-hatching) could suppress synaptic branch loss by almost 30% in *atat-2(ok2451)* animals (Fig. S2c,d). In contrast, this was not observed in *mec-17* mutants (Fig. S2e,f). Interestingly, we observed that *atat-2* mutants transitioned approximately 10 h later into L4 stage, which might explain the different dynamics of synaptic branch loss seen between these animals and those lacking *mec-17*.

These differences in the dynamics of branch loss between animals defective for *mec-17* or *atat-2* may suggest that these genes have different effects on synaptic branch stability or that they are functioning in different pathways to stabilize the PLM synaptic branches. Overall, this set of data implies that correct levels of MEC-17 and ATAT-2 are required for the stability of the PLM synaptic branch.

### MEC-17 levels control synaptic branch development

**Figure 2:**
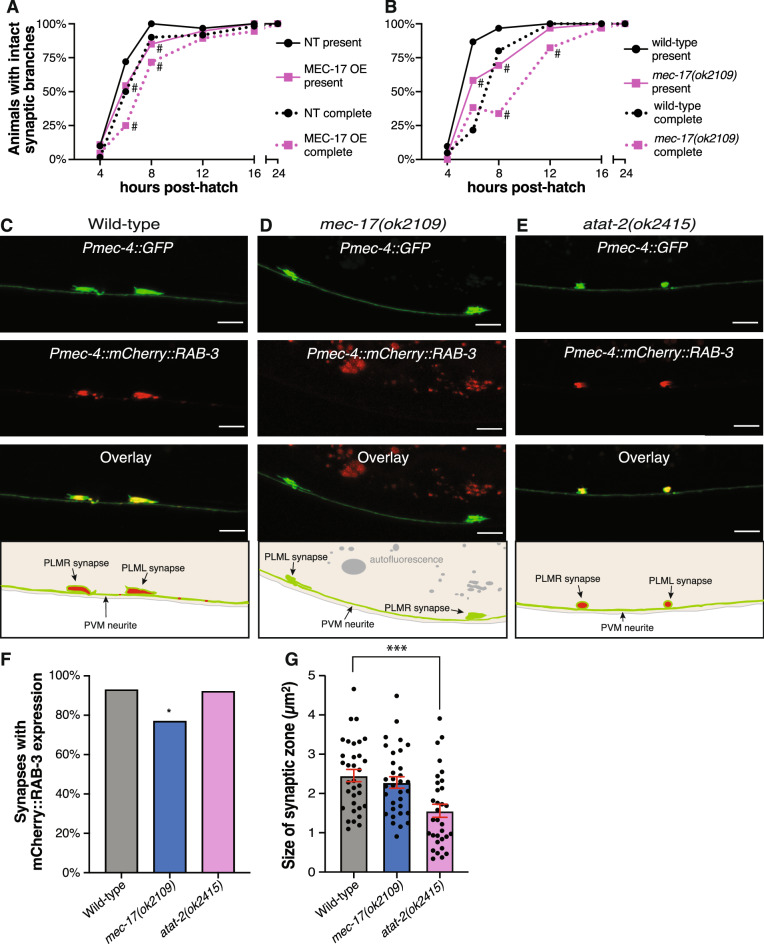
MEC-17 overexpression and loss disrupt PLM synapse development. Quantification of the number of animals displaying a branch from the main PLM axon shaft (present) and those with a branch extending into the ventral nerve cord (complete) in (**a**) animals with *mec-17* overexpression (OE) or (**b**) *mec-17* mutants. Both PLML and PLMR analyzed at 4, 6, 8, 12, 16 and 24 h post-hatch (different cohorts of animals used at each time point); n ≥ 30. # indicates P < 0.05 when comparing to the non-transgenic (NT, a) or wild-type (b) calculated from Fisher’s exact tests. (**c**) Maximum projection confocal images of the PLM synaptic sites in wild-type animals. The top panel displays the neurons expressing *Pmec-4::GFP*, the middle panel shows the pre-synaptic sites labelled with mCherry::RAB-3, the third panel is an overlay with co-localization displayed yellow-orange, and the bottom panel is a schematic of the overlay. Arrowheads point to synaptic expansions/accumulations; scale bars represent 5 μm. (**d,e**) Representative image of a *mec-17(ok2109)* and *atat-2(ok2415)* mutant animal respectively, displayed as per panel c; scale bars represent 5 μm. (**f**) Number of synapses where mCherry::RAB-3 was visualized in wild-type, *mec-17(ok2109)* and *atat-2(ok2415)* animals where n ≥ 38; P values * < 0.05 from Fischer’s exact test. (**g**) Quantification of the pre-synaptic area labelled by *Pmec-4::GFP* in wild-type, *mec-17(ok2109)* and *atat-2(ok2415)* animals; bars show mean ± SE; n ≥ 30; P values *** < 0.001 from unpaired *t*-test.

We next investigated if MEC-17 is required for the initial development of the PLM synaptic branches. Whilst the main axon shaft of PLM develops during embryogenesis^44^, the synaptic branch extends during the first larval period (L1)^11^. To quantify any temporal changes in branch development, we analyzed animals at 4, 6, 12, 16 and 24 h post-hatching for both the presence of a branch and for a complete branch (one extending into the ventral nerve cord). Compared to their non-transgenic siblings, animals with overexpression of MEC-17 displayed a significant delay in the development of the PLM branch (Fig. 2a). Deletion of *mec-17* caused a similar, but more significant delay in branch formation (Fig. 2b). This defect was most pronounced at the 8-h time point, at which 97% of wild-type animals had developed a branch (with 80% complete) but only 58% of *mec-17(ok2109)* animals had a branch (and only 38% were complete).

MEC-17 is required for the formation of the specialized 15-protofilament microtubules observed within the PLM neurons, with polymorphic microtubules consisting of between 10 and 16 protofilaments present in its absence^22,23^. In order to analyze synaptic branch development in PLM neurons lacking specialized microtubules altogether, we analyzed *mec-7/β-tubulin* null mutants, which do not form these microtubules, and compared them with *mec-12/α-tubulin* null mutants that still produce 15-protofilament microtubules^22,23^. Animals lacking MEC-7/β-tubulin displayed a significant delay in both the presence of the branch and in the appearance of a complete branch (Fig. S3a). Branch formation was not delayed in *mec-12* single mutants, whereas double mutant animals lacking both *mec-7* and *mec-12* were delayed in the development of the PLM synaptic branch to a similar level as single *mec-7* mutants (Fig. S3a). When considering complete synaptic branches, the loss of *mec-12* could suppress the delay associated with *mec-7* loss-of-function. Thus, it appears that *mec-7* is dependent on *mec-12* for this phenotype. Overall, these findings suggest that the unique 15-protofilament structure may be necessary for controlling the timing of synaptic branch development in the PLM neurons. These data also indicate that the presence of MEC-12/α-tubulin, and possibly its acetylation, may be important for controlling the timing of synaptic branch development.

The PLM synaptic branches are formed in a stereotypical position along the main axon shaft. This development is controlled by Wnt-Frizzled and Planar Cell Polarity signaling pathways that function to spatially restrict the focal accumulation of filamentous (F-) actin^45^. To examine whether MEC-17 is necessary for developmental patterning of the branch, we calculated the relative position of the branch to the cell body and axon terminus. As shown in Fig. S3b, the branch consistently extended from the main axon shaft at around two-thirds of its length, and this was unchanged by either the overexpression of MEC-17 or its loss-of-function.

To more closely study the synapses in *mec-17* and *atat-2* mutants, we visualized the localization of RAB-3 in the PLM neurons. The synaptic vesicle-associated small guanosine triphosphatase RAB-3 is the orthologue of the RAS GTPase rab3, and strongly localizes to pre-synaptic termini in discrete punctate patterns for each PLM neuron^24,46,47^. Confocal microscopy imaging of L3 stage animals expressing mCherry tagged versions of RAB-3 revealed that loss of *mec-17* significantly affected the accumulation of this pre-synaptic marker (Fig. 2c,d). Accumulation of mCherry::RAB-3 was observed in 93% (65/70) of synaptic sites in wild-type animals, but in only 77% (50/65) of synapses in *mec-17* mutants (Fig. 2f). This may suggest that MEC-17 is important for correct transport of synaptic vesicles. Interestingly, the proportion of *atat-2* mutants with accumulation of mCherry::RAB-3 at pre-synaptic sites was similar to that of wild-type animals (92%, 35/38, Fig. 2e,f), suggesting that *atat-2* may not play a significant role in axonal transport and trafficking along the synaptic branch. However, the relative area of the presynaptic site in PLM was significantly reduced in *atat-2* mutants compared to wild-type animals, but remained unchanged in *mec-17* mutants (Fig. 2g). Thus, MEC-17 and ATAT-2 may have different roles during synaptic branch development and maintenance.

These results demonstrate that correct regulation of MEC-17 is important for temporal control of synaptic branch development, as well as for the correct trafficking of pre-synaptic components. In contrast, MEC-17 does not appear to participate in the developmental patterning of the PLM synaptic branch, suggesting that it is unlikely to impact on the localized stabilization of F-actin.

### MEC-17 and ATAT-2 function largely independently from their acetylation domains to maintain synaptic branches

**Figure 3:**
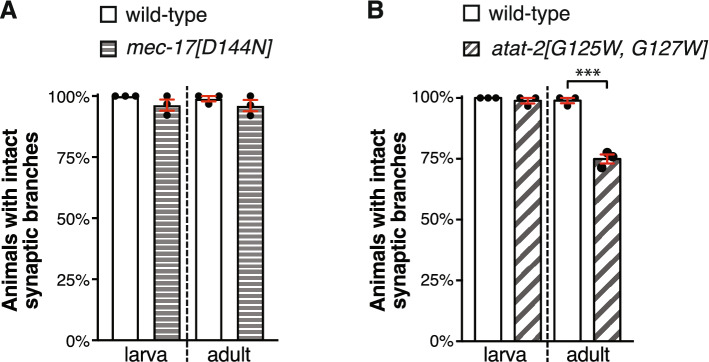
MEC-17 and ATAT-2 function largely independently from their acetyltransferase domains in maintaining synaptic branch stability. (**a**) Quantification of the number of animals with intact synaptic branches in *mec-17[D144N]* and (**b**) *atat-2[G125W, G127W]* animals compared to wild-type. Bars show mean ± SE; symbols show the mean of 3-independent experiments, each with n ≥ 21 (total n ≥ 108). *** < 0.001 from *t*-test.

To explore the role of ATAT-2 and MEC-17’s enzymatic functions in stabilizing the PLM synaptic branches, we studied the function of each proteins acetyltransferase domain. We used CRISPR/Cas9 editing to generate strains expressing versions of these proteins lacking enzymatic activity: introducing the D144N mutation into the endogenous *mec-17* gene and G125W/G127W into the endogenous *atat-2* gene^21,23,24,40^. The percentage of intact synaptic branches in MEC-17[D144N] animals was indistinguishable from the wild-type at both the L3 and A1 stages (Fig. 3a), suggesting that MEC-17 functions independently from its acetyltransferase domain to preserve the PLM synaptic branches. Conversely, animals lacking ATAT-2 enzymatic activity still displayed a significant loss of the synaptic branch (Fig. 3b). However, this was less severe compared to loss of function alleles suggesting the enzymatic activity of ATAT-2 has a minor role in synaptic branch maintenance.

**Figure 4:**
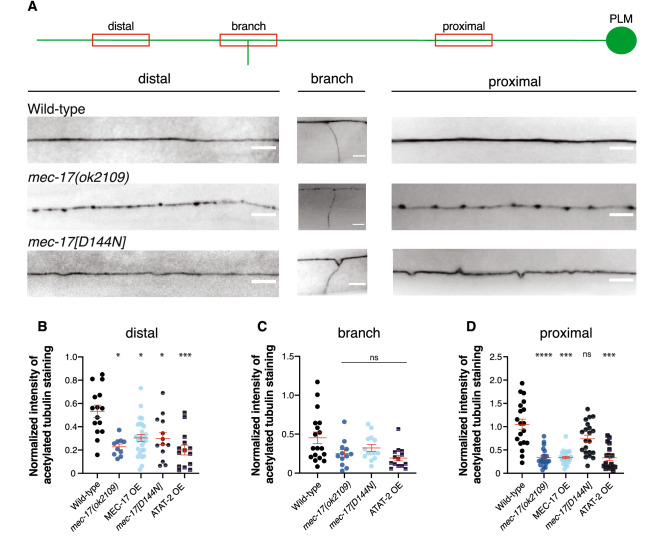
Quantification of α-tubulin acetylation levels. (**a)** Schematic depicts the PLM neuron with its proximal, branch and distal regions highlighted in red, showing the approximate locations imaged for analysis. Maximum intensity projection images show the distal, branch and proximal regions of the PLM axon in animals stained for acetylated tubulin. The panels represent the distribution of acetylated tubulin in wild-type (*zdIs5*)*, mec-17(ok2109),* and *mec-17[D144N]* animals. Scale bar is 5 µm. (**b)** Quantification of normalized intensity of acetylated tubulin staining along the distal, (**c)** branch, and (**d**) proximal regions of the PLM axon in gravid adults across the different genotypes. Background intensity was measured from pixels adjacent to the neuronal process, and normalized intensity was calculated by using the formula (I_(signal)_ – I_(background)_)/I_(background)_. Data is represented as a scatter dot plot with outliers removed and standard error of the mean marked by red lines. All genotypes are compared to wild-type (*zdIs5*). *P*-values * < 0.05, *** < 0.001, **** < 0.0001 obtained by comparing the mean rank of each distribution from the Kruskal–Wallis H test, followed by Dunne’s multiple comparisons test. Each genotype has n > 15 animals analyzed.

To examine how the modulation of *mec-17* and *atat-2* levels affected the acetylation of α-tubulin, we performed immunostaining experiments using an antibody against acetylated tubulin. Wild-type adult animals displayed consistent, uniform acetylation staining throughout the PLM neurons (Fig. 4a). Animals lacking *mec-17* displayed punctate and patchy staining along the axon (Fig. 4a), which may be a consequence of the disrupted microtubule structure previously reported in these animals^22,23^. The staining was generally fainter than wild-type animals (Fig. 4b–d) and was absent from the synaptic branch in the majority of animals (Fig. S4). The catalytic dead version of MEC-17 (D144N) was not significantly different from the wild-type in the proximal axon segment or at the branch point, and only showed a difference in acetylation levels in the distal region of the axon (Fig. 4a–d). We did not observe any staining in *atat-2* single (n > 250 animals)*,* or *atat-2; mec-17* double mutant (n > 175) animals (no data shown as no measurements could be obtained). This is largely consistent with previous findings^21,23^, and suggests that ATAT-2 is the predominant α-tubulin acetyltransferase within the PLM neurons, with MEC-17 playing a minor role.

Animals with *mec-17* or *atat-2* overexpression displayed reduced levels of acetylated α-tubulin throughout the PLM neurons (Fig. 4b–d). These observations are unexpected, as they demonstrate that overexpressing either of the acetyltransferases fails to increase the levels of α-tubulin acetylation. These findings suggest that misregulation of ATAT-2 or MEC-17 disrupts the normal acetylation process, resulting in the observed unexpected decrease in acetylated α-tubulin levels. Overall, we find no correlation between the level of α-tubulin acetylation and the loss of the PLM synaptic branches. Thus, the observed phenotypes are unlikely to merely be a consequence of disrupting the levels of α-tubulin acetylation.

**Figure 5:**
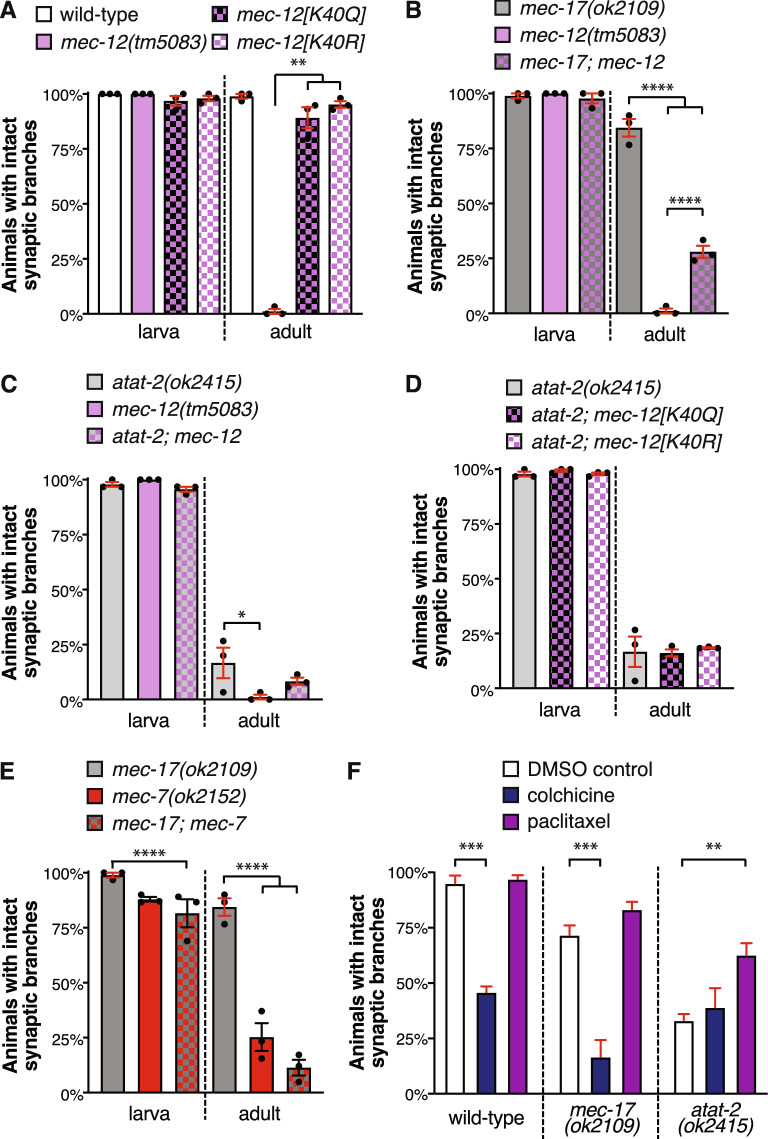
MEC-17 and ATAT-2 function independently from MEC-12/α-tubulin acetylation in maintaining synaptic branch stability. (**a**) The proportion of animals displaying intact synaptic branches in *mec-12(tm5083)*, *mec-12[K40Q]*, *mec-12[K40R]* mutants compared to wild-type. For each independent experiment n ≥ 27 (total n ≥ 87). (**b**) Quantification of animals with intact synaptic branches in *mec-17(ok2109)* and *mec-12(tm5083)* single and double mutant animals. For each independent experiment, n ≥ 29 (total n ≥ 89). (**c**) Quantification of animals with intact synaptic branches in *atat-2(ok2415)* and *mec-12(tm5083)* single and double mutant animals. For each independent experiment, n ≥ 23 (total n ≥ 80). (**d**) The percentage of intact synaptic branches in animals carrying the *atat-2(ok2415)* mutant alone, or in combination with the *mec-12[K40Q]* or *mec-12[K40Q]* mutations*.* For each independent experiment n ≥ 29 (total n ≥ 89). (**e**) Quantification of animals with intact synaptic branches in *mec-17(ok2109)* and *mec-7(ok2152)* single and double mutant animals. For each independent experiment, n ≥ 30 (total n ≥ 90). Bars show mean ± SE; *P* values * < 0.05, ** < 0.01, *** < 0.001, **** < 0.0001 from one-way ANOVA with Tukey’s post-hoc test. (**f**) The percentage of wild-type, *mec-17(ok2109)* and *atat-2(ok2415)* animals (transgenic) with intact synaptic branches when treated with 1% DMSO (control, white bars), 0.5 mM colchicine (dark blue bars), or 10 μM paclitaxel (purple bars). For three independent experiments n ≥ 20 (total n ≥ 62). Bars show mean ± SE; *P* values ** < 0.01, *** < 0.001 from one-way ANOVA with Tukey’s post-hoc test.

In order to further investigate the role of α-tubulin acetylation, we studied animals carrying different versions of MEC-12, the sole *C. elegans* α-tubulin protein containing a K40 acetylation site^48^. Using CRISPR/Cas9 editing, we generated strains carrying versions of MEC-12 that mimic pan-K40 acetylation (MEC-12[K40Q]) or that cannot be acetylated (MEC-12[K40R])^20^ and compared these with *mec-12(tm5083)* null mutants. Consistent with the findings of Chen et al*.*^7^, loss of *mec-12* caused a severe synaptic branch defect, with only 6% of animals maintaining both their synaptic branches into adulthood (Fig. 5a). Animals carrying either the K40Q or K40R versions of MEC-12 displayed wild-type levels of branches at both larval and adult stages (Fig. 5a). These data imply that MEC-12 is required for the stability of the PLM branches, but that it functions independently from its K40 acetylation site in this context. Overexpression of *mec-17* in the *mec-12[K40Q]* and *mec-12[K40R]* mutant backgrounds was still sufficient to disrupt the PLM synaptic branches (Fig. S5a), providing further evidence in support of a non-enzymatic role for MEC-17 in branch stability.

### MEC-17 functions independently from MEC-12/α-tubulin in synaptic branch stability

Since MEC-17 appears to function independently from the acetylation of MEC-12/α-tubulin, we next sought to investigate whether it is still dependent on the tubulins for the maintenance of the PLM synaptic branches. Surprisingly, animals lacking both *mec-12* and *mec-17* partially suppressed the *mec-12* single mutant synaptic branch phenotype (Fig. 5b). This suggests that the *mec-12* mutant phenotype is partly dependent on *mec-17* and that these two genes may not function in the same pathway. Interestingly, despite a similar trend towards a less severe defect, double mutants between *atat-2* and *mec-12* were not significantly different from *mec-12* single mutants (Fig. 5c), suggesting that *atat-2* (but not *mec-17*) functions in the same genetic pathway as *mec-12*. The penetrance of the defect in *atat-2* mutants was unaffected by mutating the K40 site of *mec-12* to mimic either pan-K40 acetylation or to inhibit acetylation (Fig. 5d). Thus, similar to MEC-17, ATAT-2 functions independently of the acetylation of MEC-12 to stabilize the PLM synaptic branches.

We next sought to test the importance of *mec-7/β-tubulin* for PLM synaptic branch stability and interaction with *mec-17*. Consistent with previous reports^7^, disruption of *mec-7* caused the loss of synaptic branches in the majority (75%) of adult animals (Fig. 5e). We also observed a reduction in the proportion of intact branches at the L3 stage in *mec-7* loss-of-function mutants, with 12% of animals lacking a branch (Fig. 5e). Animals lacking both *mec-7* and *mec-17* displayed similar levels of intact synaptic branches to the single *mec-7* mutants at both L3 and A1 stages (Fig. 5e), suggesting that *mec-17* functions in the same genetic pathway as *mec-7*.

Animals lacking both *mec-7* and *mec-12* had a significantly higher percentage of intact branches than either of the single mutants (Fig. S5b). At A1 stage, 51% of double mutants had intact branches, compared to 25% in *mec-7* single mutants and 1% in *mec-12* single mutants. At the L3 stage, 98% of the double mutants had intact synaptic branches, suppressing the defect observed in *mec-7* single mutants. Thus, loss-of-function mutations in either *mec-7* or *mec-17* can inhibit the defects associated with *mec-12*.

In sum, these data provide further evidence for MEC-17 functioning independently from MEC-12 in synaptic branch stability. Instead, they point towards an interaction with MEC-7/β-tubulin. Alternatively, *atat-2* appears to function in the same genetic pathway as *mec-12*. In addition, our data establishes that MEC-17 and ATAT-2 stabilize the PLM synaptic branches independently from α-tubulin acetylation.

### Microtubule destabilization can disrupt synaptic branch stability

In order to gain insight into how *mec-17* and *atat-2* impact microtubule stability, we treated animals with microtubule stabilizing (paclitaxel) and de-stabilizing (colchicine) drugs^24,49^. First, to assess the impact of destabilization, animals were grown on a low concentration of colchicine, which promotes microtubule depolymerization by binding to free tubulin^50^. Colchicine induced loss of the synaptic branches in more than 50% of wild-type adult animals (Fig. 5f), indicating that destabilization of the microtubule network by promoting depolymerization can cause the loss of the PLM synaptic branches. The penetrance of the defect in *mec-17* mutants was significantly worsened with colchicine treatment, but no change was observed in *atat-2* animals (Fig. 5f). Next, to evaluate the effect of microtubule hyper-stabilization, animals were grown on a low concentration of paclitaxel, which suppresses microtubule dynamics by binding to microtubule bundles^50^. Treatment with paclitaxel led to a significant rescue of the synaptic branch defect in *atat-2* mutants, while wild-type and *mec-17* mutant animals were unaffected (Fig. 5f).

It is curious that paclitaxel could not significantly rescue the defect in *mec-17* mutants, and that colchicine could not exacerbate the *atat-2* phenotype. These findings may be due to the low penetrance of the defect in *mec-17* mutants and the strong defect in *atat-2* mutants that may be difficult to modulate with these drugs. However, as colchicine treatment does not worsen the *atat-2* mutant defects, and paclitaxel can rescue them, it may suggest that microtubule stability is already severely impaired in *atat-2* mutants. Thus, ATAT-2 may predominately function to stabilize microtubules, while MEC-17 may have additional roles outside of microtubule stability. It is also possible that these drugs interact differently with the microtubule network than MEC-17 and ATAT-2. Nevertheless, these data provide support to the hypothesis that the loss of MEC-17 and ATAT-2 destabilizes the microtubule network and suggest that normal microtubule dynamics may be important for maintaining synaptic branches.

### MEC-17 and ATAT-2 function together with ZYX-1 in the maintenance of synaptic branches

**Figure 6:**
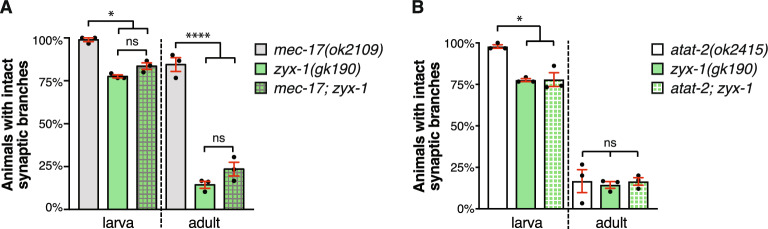
*mec-17* and *atat-2* function in the same genetic pathway as *zyx-1*. (**a**) Quantification of animals with intact synaptic branches in *mec-17(ok2109)* and *zyx-1(gk190)* single and double mutants. Bars show mean ± SE; symbols show the mean of three-independent experiments, each with n ≥ 29 (total n ≥ 88). (**b**) Analysis *atat-2(ok2415)* and *zyx-1(gk190)* single and double mutant animals. For each independent experiment, n ≥ 28 (total n ≥ 87). P values ns > 0.05, * < 0.05, **** < 0.0001 from one-way ANOVA with Tukey’s post-hoc test.

Previous research demonstrated that the focal adhesion protein ZYX-1 functions in the maintenance of PLM synaptic branches^51^. To determine if *mec-17* and *atat-2* function in the same genetic pathway as *zyx-1*, we quantified synaptic branch defects in single and double mutant animals. Analysis of single mutant animals revealed that mutation of *zyx-1* caused a progressive loss of the PLM synaptic branches (Fig. 6a). The phenotype observed in animals carrying mutations in both *mec-17* and *zyx-1* was not worse than the single *zyx-1* mutants (Fig. 6a). Thus, *mec-17* may function in the same genetic pathway as *zyx-1* to maintain the PLM synaptic branches. To assess whether *atat-2* might also genetically interacts with *zyx-1* in this context, we analyzed double mutants between *atat-2* and *zyx-1*. As shown in Fig. 6b, *atat-2; zyx-1* double mutants did not present a worsening of the defect compared to the respective single mutants. Thus, *atat-2* appears to function in the same genetic pathway as *zyx-1* to regulate synaptic branch stability.

Overall, our study reveals the importance of correct levels of MEC-17 and ATAT-2 for the stability of neuronal architecture. Our genetic analysis uncovered novel interactions for both *mec-17*, *atat-2* with *zyx-1*, a gene previously associated with actin remodelling under mechanical stress conditions^52–55^. Thus, it is possible that interaction between microtubules and the actin network may be important for the preservation of neuronal structure over an animals’ lifetime.

## Discussion

Previous research has provided significant links between microtubule dynamics and synaptogenesis. For example, the formation of synaptic boutons in *Drosophila melanogaster* requires a transition from dynamic microtubules in growth cones to stable loop-like structures that promote synaptic formation^56–58^. Interestingly, mutation of *mec-12/α-tubulin* or *mec-7/β-tubulin* in *C. elegans* results in smaller pre-synaptic varicosities^7^, which may suggest that similar mechanisms may also occur in the nematode. Our data adds further support to the importance of microtubule dynamics in this process, revealing that correct levels of both MEC-17 and ATAT-2 are required for temporal control of synaptic branch formation, and the correct localization of pre-synaptic components.

Misregulation of MEC-17 and ATAT-2 disrupts the stability of the microtubule network, resulting in loss of the PLM synaptic branch. Through the analysis of acetylated α-tubulin and the importance of the catalytic domains of ATAT-2 and MEC-17, our data implies that these proteins function mostly independently from their ability to acetylate α-tubulin. In contrast, a recent study supports the importance of α-tubulin acetyltransferases in maintaining the PLM synaptic branches^40^. However, compared to our study, Borgen et al*.* observed a moderate loss of synaptic branches in both *atat-2* and *mec-17* mutants. We speculate that these differences may result from disparities in genetic backgrounds or experimental set-ups. Indeed, we quantified the presence of synaptic branches between 52 and 60 h (A1), while Borgen et al*.* appear to have made their measurements at 48 h. During these 4–12 h, we show that branches are progressively lost (see Fig. 1h), which may account for the observed differences in penetrance. Additionally, we observed a more significant rescue of the phenotype when treating worms with the microtubule stabilizing drug paclitaxel, which likely resulted from differences in concentration (10 μM in our study versus 2 μM by Borgen et al*.*). In direct contrast to our study, Borgen et al*.* concluded that ATAT-2 functions through its acetyltransferase domain to maintain the synaptic branches. Although it is not completely clear how we arrived at different conclusions, we believe that our use of a CRISPR-Cas9 edited endogenous *atat-2* (rather than the use of an extrachromosomal array, which likely produces much higher levels of ATAT-2), provides strong support for our conclusions.

The synaptic branches of the PLM neurons appear to be particularly vulnerable to changes in the levels of MEC-17 and ATAT-2 proteins. Based on the position of the branches near the lateral surface of the cuticle, and the typical sinusoidal movements of *C. elegans*, it seems plausible that this susceptibility is a result of the mechanical stress associated with the continuous dorsal–ventral bending during locomotion. This is strongly supported by our paralysis experiments, which revealed that suppression of animal movement could prevent synaptic branch loss. Indeed, disruptions to microtubules and other components of the cytoskeleton within the *C. elegans* mechanosensory neurons have been reported to induce buckling of the axons and overt degeneration upon animal movement^24,59–61^. Furthermore, the disruptions in protofilament number and MT structure caused by *mec-17* loss of function^22,23^ may weaken the branch and make it more sensitive to mechanical stress.

How MEC-17 and ATAT-2 function to impart stress-resistance remains to be determined. Our immunostaining data largely mirrors that of previously publications^20,21,23^, and provides further support for ATAT-2 being the predominant acetyltransferase enzyme in *C. elegans*. However, our data conclusively demonstrates that these molecules function independently from the acetylation of MEC-12/α-tubulin. MEC-17 has previously been proposed to function as a structural component within the microtubules^23^, but the precise details of how it functions in this regard are yet to be determined. It is plausible that it has a similar structural role, possibly together with ATAT-2, in mediating synaptic branch stability. Recent studies have suggested that luminal proteins form a molecular network that regulates microtubule stability from within^62^. This could provide an explanation for how MEC-17 and ATAT-2 are functioning in the context of synaptic branch stability where too much or too little of these proteins may lead to changes in either the architecture or stability of microtubules in the branch independent of microtubule acetylation. The genetic interaction between *mec-17* and *mec-7/β-tubulin* may support this hypothesis, as it might point towards an important relationship between these molecules for microtubule stability. Alternatively, MEC-17 and ATAT-2 might interact with other tubulin molecules known to be present in the PLM neurons (α-tubulins TBA-1, TBA-2 and TBA-6, and β-tubulins TBB-1 and TBB-2)^63^, or perhaps, molecules outside of the microtubule network.

Interactions between microtubules and the actin network are increasingly being recognised as essential facets controlling cytoskeletal dynamics. The coordinated activities of these major cytoskeletal components have been best described during neuronal development^64^. Axon elongation from a growth cone is achieved by local depolymerization of the actin meshwork allowing polymerizing microtubules to protrude towards the leading edge and thereby extend the axon shaft^65^. Our discovery of possible interactions between *mec-17* and *zyx-1*, and between *atat-2* and *zyx-1* in the protection of the PLM synaptic branches suggests that microtubule-actin interactions are also crucial for the maintenance of neuronal structure. Various studies have demonstrated the importance of zyxin for actin remodelling, particularly under conditions of mechanical stress^52–55^. The likely function of *zyx-1* in the same genetic pathway as both *mec-17* and *atat-2* may therefore suggest that actin-microtubule interactions are essential for the preservation of synaptic branches.

In conclusion, this work establishes MEC-17 and ATAT-2 as essential mediators of synaptic development and maintenance. These proteins function independently from their well-known roles in acetylating α-tubulin and appear to function in different pathways in this context.

## Methods

### *C. elegans* strains and genetics

Maintenance, crosses and other genetic manipulations were all performed via standard procedures^42^. Hermaphrodites were used for all experiments, and unless otherwise specified were grown at 20 °C on nematode growth medium (NGM) plates seeded with OP50 *E. coli*. The *atat-2(ok2415), atat-2[G125W, G127W], mec-12[K40Q], mec-12[K40R], mec-12(tm5083), mec-17[D144N], mec-17(ok2109)* and, *zyx-1(gk190)* mutant strains were used, together with the following transgenes: *cjnEx036*/*cjnEx038(Pmec-4::mec-17* [10 ng/µL]; *Pmyo-2::mCherry*), *cjnEx068*/*cjnEx069*/*cjnEx070*(*Pmec-4::atat-2* [10 ng/µL]; *Pmyo-2::mCherry*), *jsIs37(Pmec-7::snb-1::GFP)*, *uIs115(Pmec-17::tagRFP)*^66^, *vdEx539(Pmec-4::mec-17* [10 ng/µL]; *Plad-2::mCherry*), *zdIs5(Pmec-4::GFP).* A full list of strains is provided in Supplementary Table 1.

In vitro transcription was used to generate *unc-54* double-stranded RNA, as we have previously described^24^. Briefly, P0 animals were microinjected and their paralyzed F1 progeny were analyzed at the A1 stage.

### Analysis of neuronal morphology

Animals were synchronized using a hatch-off method^49^, whereby newly hatched larvae (0–30 min post-hatch) were collected from plates containing only eggs and transferred to new plates. L3-stage animals were scored 26–28 h post-hatch, while A1-stage animals were scored ~ 24 h after isolating them at L4 stage. For the analysis of PLM branch development in Fig. 2a, animals were collected within 15 min post-hatch and grown at room temperature (~ 22 °C).

Animals were immobilized in 0.05% tetramisole hydrochloride on 4% agar pads and imaged using a Zeiss Axio Imager M2 microscope with an Axiocam 506 mono camera and ZEN pro software. A synaptic branch was considered intact if it remained continuous from the main PLM axon to the ventral nerve cord. The synaptic branches of both PLM neurons were analyzed in each animal and animals were scored as having intact synaptic branches only if both branches were intact. For analysis of branch development in Fig. 2a and 2b, a branch was qualified as ‘present’ if it extended more than ~ 2 µm from the main axon shaft towards the ventral nerve cord, and as ‘complete’ if it extended to the ventral nerve cord.

The positions of the PLM synaptic branches shown in Fig. 2b, were calculated using the NeuronJ plugin in ImageJ 1.52 h. A line was traced from the edge of the soma to the branch point and this length was then divided by the total length of the axon (measured from the soma edge to the axon terminus).

### RNA extraction and RT-qPCR

Total RNA was extracted from L4 staged worms synchronized by bleaching. Firstly, animals were snap-frozen in liquid nitrogen and underwent seven cycles of thawing and refreezing to promote cuticle breakage. Total RNA was then extracted using the Trizol (Ambion)/chloroform method followed by the RNeasy kit (Qiagen) using the manufacturers’ guidelines. Total RNA was reverse transcribed using the ImPromII kit (Promega). qPCR was performed on a Light Cycler 480 II (Roche) with SYBR GREEN (LifeScience). Housekeeping genes *ama-1*, *cdc-42*, and *pmp-3* were used to normalize the data. Primers were designed to cover an exon-exon junction to avoid the amplification of DNA contaminants.

### Confocal microscopy

Animals were immobilized in 0.05% tetramisole hydrochloride on 2–3% agarose pads and analyzed using a Zeiss LSM980 with Airyscan 2 confocal microscope (Zeiss Group, Oberkochen, Germany) equipped with ZEN 2 software. All images were taken using the Airyscan Multiples (MPLX)-Super Resolution (SR)-4Y mode and C Plan Apochromat 63x/1.4 objective. Bidirectional confocal imaging with 2 × averaging was conducted for all animals using a 488 nm solid state laser (0.8% power) to visualize GFP and a 561 nm laser (5.0% power) for the mCherry fluorophore. An optimal z-step size of 0.15 μm was used for all z-stacks with a zoom factor of 2, obtaining a consistent image size of 1584 × 1584 pixels. For the qualitative analysis of RAB-3 at pre-synaptic sites, images were studied using Fiji/Image J (version 2.1.0/1.53c). All animals were imaged at 26–28 h post hatch to allow the analysis of RAB-3 localization in intact synaptic branches. The size of synaptic sites was calculated from maximal projections of the obtained z-stacks with Fiji/Image J (version 2.1.0/1.53c), with the synaptic regions defined by hand.

### Immunostaining of *C. elegans*

*C. elegans* were fixed in 2% PFA-methanol fixative and incubated at -80ºC overnight. Animals were then incubated at 4ºC overnight in mouse anti-acetylated tubulin primary antibody (Sigma T6793), at a dilution of 1:100 in 0.5% PBST, and subsequently in goat anti-mouse Alexa Fluor 488 secondary antibody (Invitrogen A11001), at a dilution of 1:100 in 0.5% PBST at 4 ºC overnight. Immunostained animals were mounted on glass slides using a mounting medium consisting of 0.1 g *N*-Propyl Gallate, 3.5 mL Glycerol, and 1.5 mL of 100 mM Tris pH 9.5 in 0.5% PBST. Slides were imaged on an Olympus IX73 inverted epifluorescence microscope, using a 100×, 1.4 N.A oil objective, with an X-cite Mercury vapour short arc lamp at 100% iris setting, and an Andor EMCCD camera operated at gain 50 and exposure 100 ms. Images were generated by combining successive z-stacks using the maximum intensity projection and inverted display features in FIJI (ImageJ) image analysis software. Average intensity of staining along the proximal, branch and distal processes of the PLM neuron were measured using the ‘Segmented Line’ and ‘Measure’ functions in FIJI. Background intensity was measured from pixels adjacent to the neuronal process, and normalized intensity was calculated by using the formula (I_(signal)_ – I_(background)_)/I_(background)_. Genotypes are compared to wild-type (*zdIs5*) using the Kruskal–Wallis non-parametric test and Dunne’s multiple comparisons test.

### Drug treatment

Animals were grown on NGM plates containing either colchicine (Sigma-Aldrich) or paclitaxel (Sigma-Aldrich) dissolved in DMSO as previously described^24,49^. Control animals were grown on NGM plates containing 1% DMSO, a concentration far exceeding the maximum amount used in the drug treatment plates (> 0.05%). L4 stage animals were transferred to the drug plates and their F1 progeny scored at A1 stage. For analysis of the effects of paralysis on the synaptic branch phenotypes, animals were immobilized in 0.05% tetramisole hydrochloride on 2–3% agarose pads for the time periods shown in Figure S2c–f.

### Statistical analysis

Statistical analysis was performed using GraphPad Prism 8. Two-way comparisons were performed using unpaired *t*-tests, and ANOVA was used for comparing groups with more than two samples, followed by Tukey’s multiple comparisons post-hoc tests. Chi-square tests were used to compare the distribution of phenotypes between populations.

## Supplementary Information

Supplementary Information.

## Data Availability

All data generated or analysed during this study are included in this published article (and its supplementary information files).
