# Supplementary material for: Synaptic branch stability is mediated by non-enzymatic functions of MEC-17/αTAT1 and ATAT-2
Source: Sci Rep. 2022 Aug 17;12:14003. doi: 10.1038/s41598-022-18333-2 (PMC9385713; doi:10.1038/s41598-022-18333-2)
Supplement: Supplementary file 1 — Supplementary Information. [file 41598_2022_18333_MOESM1_ESM.pdf]

Supplementary Figure 1. PLM synaptic branch defects and quantification of gene expression levels. Panels provide ventral view examples of the phenotypes observed in animals carrying the *zdIs5(Pmec-4::GFP)* transgene and overexpression of MEC-17: (**a**) branch thinning; (**b**) remnants on the main axon shaft; (**c**) remnants on the pre-synaptic side; (**d**) remnants on both sides. White arrows point to intact branches; magenta arrows to thin branches; arrowheads to branch remnants; boxes highlight the enlarged images shown to the right of each larger image. Scale bars represent 50 µm. (**e**) Relative changes in gene expression across the different strains with *mec-17* overexpression, or (**f**) *atat-2* overexpression. Columns show the mean ± SE for three replicate experiments. (**g**) Quantification of the number of animals with intact synaptic branches in *mec-17(ok2109)* (left two bars) or *atat-2(ok2415)* (right two bars) mutant animals without (-) a cell-autonomous rescue construct (*Pmec-4::mec-17* or *Pmec-4::atat-2*) or with (+) the construct. Bars show mean ± SE; symbols show the mean of three-independent experiments, each with $n \geq 30$ (total $n \geq 90$). *P* values ** < 0.05, from one-way ANOVA with Tukey's post-hoc test.

Supplementary Figure 2. Immobilization can suppress the loss of PLM synaptic branches. (**a**) Quantification of the proportion of animals with intact synaptic branches in *mec-17* overexpression, *mec-17(ok2109)*, and *atat-2(ok2415)* backgrounds treated with an empty vector (-) or with *unc-54* RNAi (+). Bars show mean ± SE; symbols show the mean of three-independent experiments, each with $n \geq 30$ (total $n \geq 90$). (**b**) Proportion of intact synaptic branches in *atat-2(ok2415)* animals untreated or treated with anesthetic for 6 hours; $n \geq 23$ (**c**) Quantification of intact synaptic branches in untreated *atat-2(ok2415)* animals compared to those treated with anesthetic 39-45 h post-hatch for three independent experiments $n \geq 20$ (total $n \geq 47$) or (**d**) 48-54 h post-hatch ($n \geq 15$; total $n \geq 46$). (**e**) Proportion of *mec-17(ok2109)* animals with intact synaptic branches when untreated or

treated with anesthetic 39-45 h post-hatch (n ≥ 20; total n ≥ 40) or (**f**) 48-54 h post-hatch (n ≥ 20; total n ≥ 40). *P* values ** < 0.05, *** < 0.001 from one-way ANOVA with Tukey's post-hoc test.

Supplementary Figure 3. Analysis of PLM synaptic branch development. (**a**) Quantification of the proportion of animals displaying a branch from the main PLM axon shaft (present) and those with a branch extending into the ventral nerve cord (complete). Both PLML and PLMR analyzed at 4, 6, 8, 12, 16 and 24 h post-hatch (different cohorts of animals used at each time point); n ≥ 30. Table below graph indicates statistical differences compared to wild-type animals calculated from Fisher's exact tests. (**b**) Quantification of the PLM branch position 8 h post-hatch. Position calculated relative to the PLM soma and axon terminus. Circles represent individual axons analyzed; n ≥ 38; mean ± SE shown in red; no significant differences observed from one-way ANOVA with Tukey's post-hoc test.

Supplementary Figure 4. Analysis of PLM synaptic branch in animals stained for α-tubulin acetylation. Proportion of animals with acetylated tubulin staining in animals with either normal synaptic branches, ectopic branches or no branches quantified across the genotypes displayed; n >15 animals analyzed for each genotype.

Supplementary Figure 5. Analysis of genetic interactions between *mec-12* and either *mec-17* or *mec-12*. (**a**) The percentage of intact synaptic branches for animals with *mec-17* overexpression, *mec-12[K40Q]*, and *mec-12[K40Q]*, compared with *mec-17* overexpressed in the *mec-12[K40Q]*, and *mec-12[K40Q]* backgrounds. Bars show mean ± SE; symbols show the mean of 3-independent experiments, each with n ≥ 30 (total n ≥ 30). (**b**) Quantification of intact synaptic branches in *mec-7(ok2152)* and *mec-12(tm5083)* single

mutants, and in animals carrying both these mutations. For each independent experiment, n ≥ 25 (total n ≥ 85). *P* values * < 0.05, ** < 0.01, *** < 0.001 from one-way ANOVA with Tukey's post-hoc test.

Supplementary Figure 1

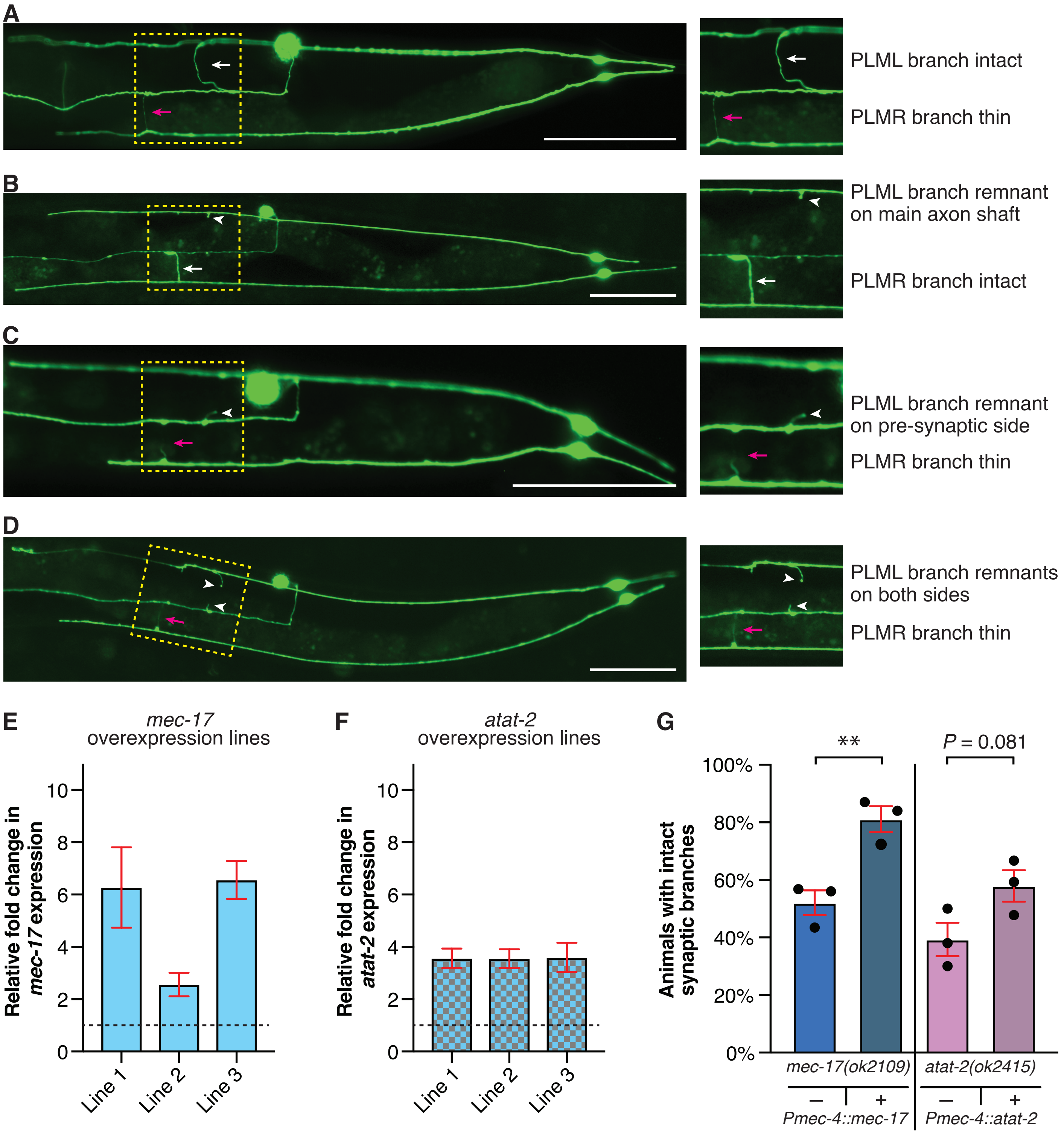

Supplementary Figure 2

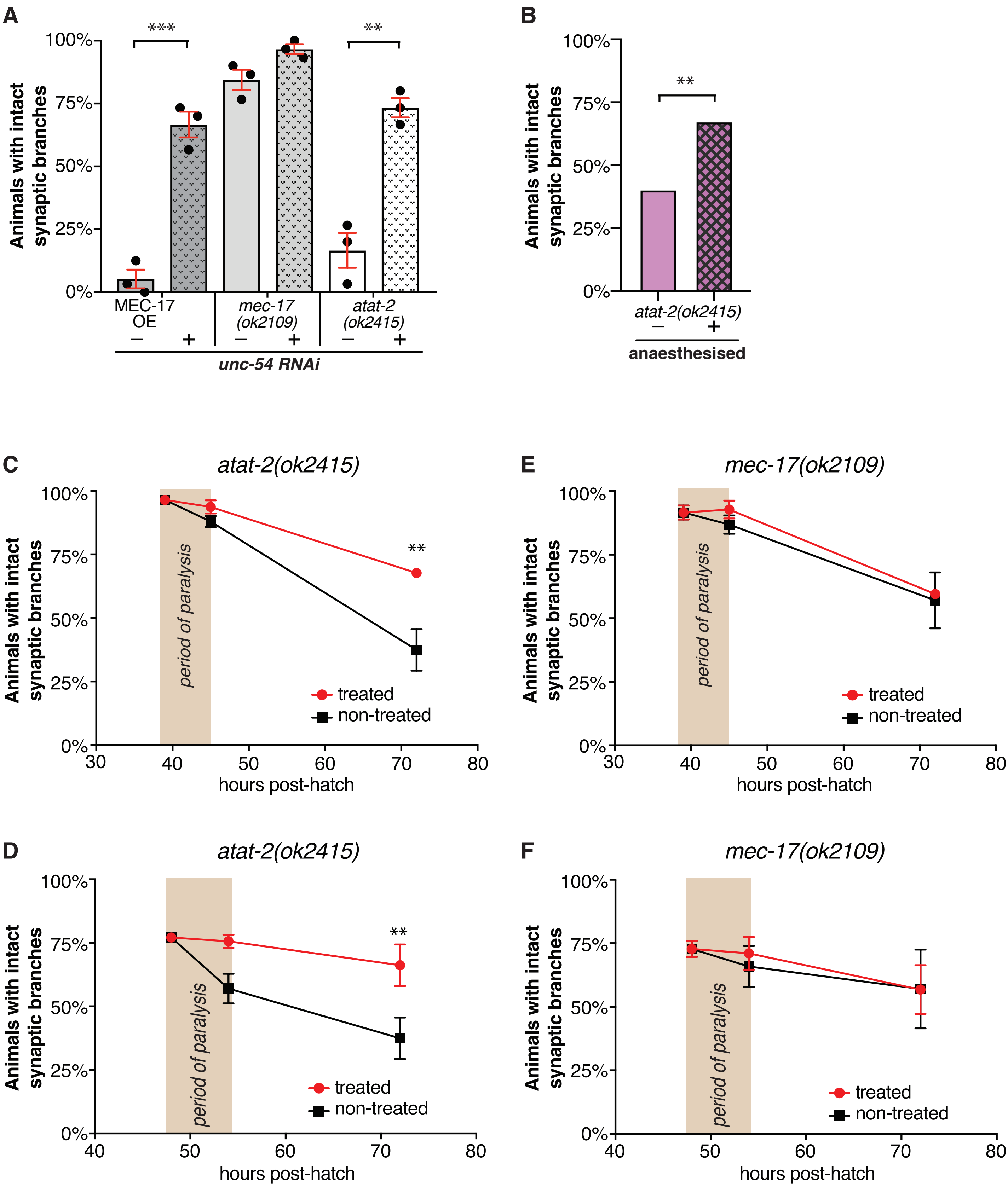

Supplementary Figure 3

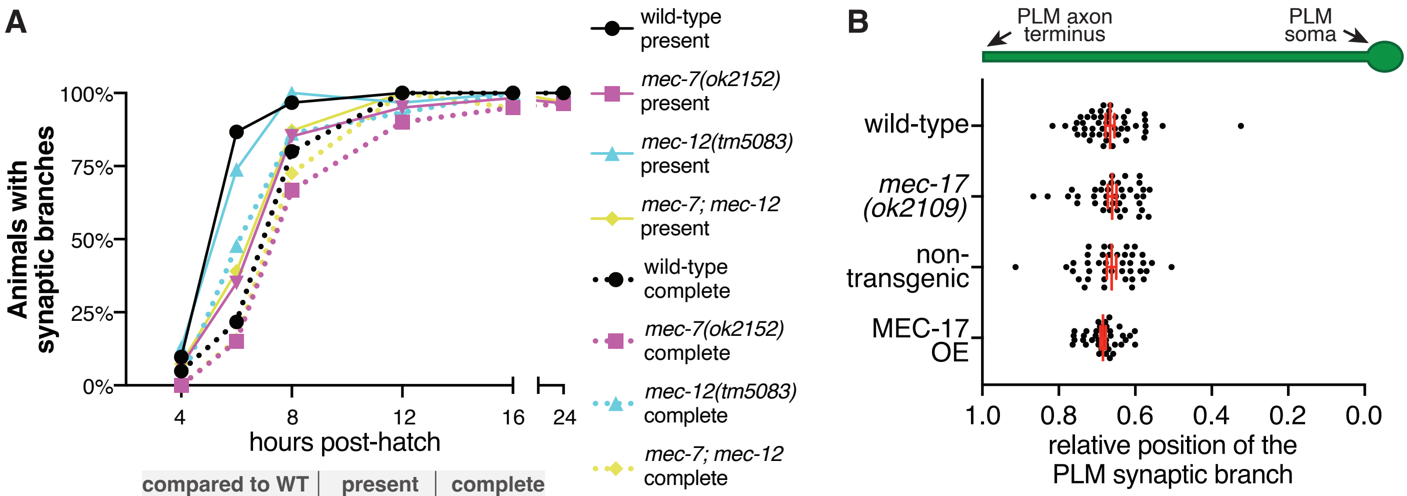

compared to WT	present	complete
mec-7	6 h, 8 h	12 h
mec-12	*NS*	6 h
mec-7; mec-12	6 h, 8 h	*NS*

Supplementary Figure 4

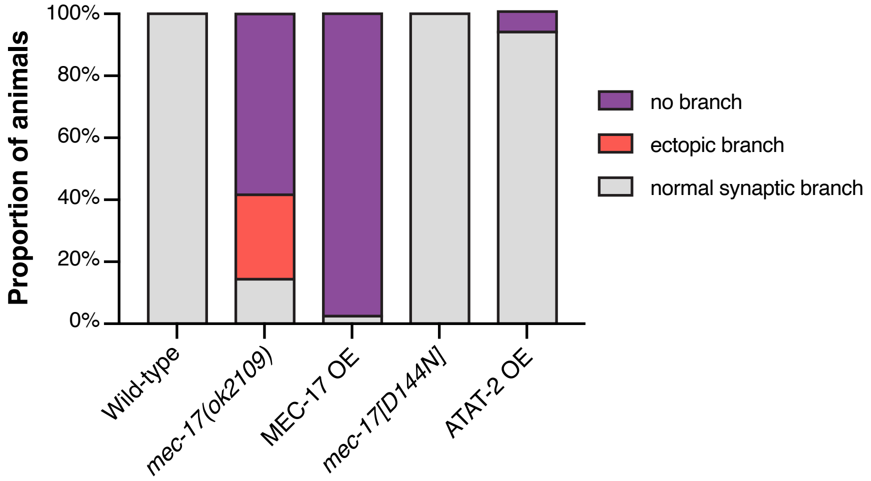

Supplementary Figure 5

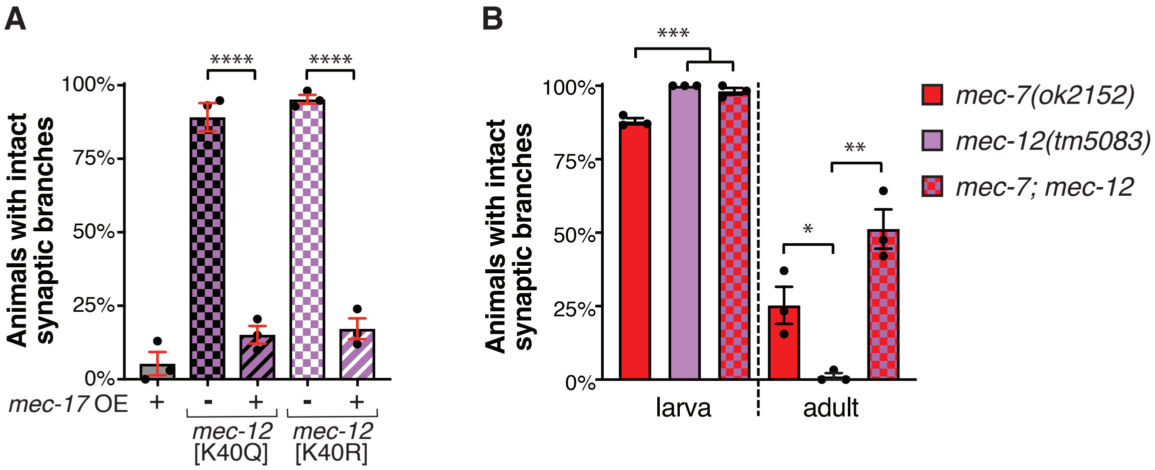

Supplementary Table 1. Strains used in this study.

Strain	Genotype	Origin
**QH3135**	*zdIs5(Pmec-4::GFP)*	Massimo Hilliard
**BXN001**	*zdIs5 I ; vdEx539(Pmec-4::mec-17; Plad-2::mCherry)*	This study
**BXN205**	*zdIs5 I ; cjnEx038(Pmec-4::mec-17; Pmyo-2::mCherry)*	This study
**BXN232**	*zdIs5 I ; cjnEx036(Pmec-4::mec-17; Pmyo-2::mCherry)*	This study
**BXN258**	*zdIs5 I; cjnEx068(Pmec-4::atat-2; Pmyo-2::mCherry)*	This study
**BXN259**	*zdIs5; cjnEx069(Pmec-4::atat-2; Pmyo-2::mCherry)*	This study
**BXN260**	*zdIs5 I; cjnEx070(Pmec-4::atat-2; Pmyo-2::mCherry)*	This study
**QH3568**	*zdIs5 I; mec-17(ok2109) IV*	Massimo Hilliard
**QH3574**	*zdIs5 I; atat-2(ok2415) X*	Massimo Hilliard
**QH3623**	*zdIs5 I; mec-17(ok2109) IV; atat-2(ok2415) X*	Massimo Hilliard
**BXN507**	*cjnEx036; jsIs37(Pmec-7::snb-1::GFP); uIs115(Pmec-17::tagRFP)*	This study
**BXN492**	*cjnEx036; vdEx262(Pmec-4::mCherry::rab-3; Punc-122::GFP); zdIs5*	This study
**BXN789**	*zdIs5 I; atat-2(syb2451) III*	This study
**BXN733**	*zdIs5 I; mec-17(syb1496) IV*	This study
**BXN633**	*zdIs5 I; mec-12(tm5083) III*	This study
**BXN718**	*zdIs5 I; mec-12(syb1477) III*	This study
**BXN722**	*zdIs5 I; mec-12(syb1498) III*	This study
**BXN705**	*zdIs5 I; mec-12(tm5083) III, mec-17(ok2109) IV*	This study
**BXN784**	*zdIs5 I; mec-12(tm5083) III; atat-2(ok2415) X*	This study
**BXN703**	*zdIs5 I; mec-12(tm5083) III, mec-7(ok2152) X*	This study
**BXN694**	*zdIs5 I; zyx-1(gk190) II*	This study
**BXN528**	*zdIs5 I; zyx-1(gk190) II; mec-17(ok2109) IV*	This study
**BXN786**	*zdIs5 I; mec-12(syb1498) III; atat-2(ok2415) X*	This study
**BXN785**	*zdIs5 I; mec-12(syb1477) III; atat-2(ok2415) X*	This study
